# Sclerosing Epithelioid Fibrosarcoma of the Coccyx: A Case Report and Review of Literature

**DOI:** 10.7759/cureus.2407

**Published:** 2018-04-02

**Authors:** Ajay Popli, Rajat Mahajan, Tarush Rustagi, Saransh Gupta, Vivek Verma, Hemant Gupta

**Affiliations:** 1 Spine, Max Hospital Vaishali; 2 Department of Spine Surgery, Indian Spinal Injuries Center, New Delhi, IND; 3 Department of Spine Surgery, Indian Spinal Injuries Centre, New Delhi, IND; 4 Orthopedic Oncology, Max Hospital Vaishali; 5 Surgery, Max Hospital Vaishali

**Keywords:** coccydynia, fibrosarcoma

## Abstract

Coccydynia in adult patients is not uncommon and is frequently neglected. Coccydynia is mostly associated with fall on buttocks. In long-standing cases, coccydynia can be debilitating. Rarely coccydynia can be due to more sinister causes and surgeons should be aware of all differential diagnosis. We present a case of an elderly female who presented with a complaint of pain over coccyx which was not subsiding with conventional treatment methods. Biopsy was done and a diagnosis of sclerosing epitheloid fibrosarcoma was made. We describe an unusual case of coccydynia secondary to this tumour with the histopathology finding and surgical management.

## Introduction

Sclerosing epithelioid fibrosarcoma (SEF) is an uncommon but aggressive sarcoma [1]. It is a soft tissue tumor with a predilection for extra-osseous sites. It usually involves one of the extremities or the torso and clinically presents as a deep soft tissue mass [2]. We here describe an unusual case of coccygeal SEF with the treatment details. SEF is a benign looking tumor morphologically but is aggressive and metastasis may be common finding on presentation. Mortality with SEF can range from 25 to 57%.

## Case presentation

Clinical history

**Figure 1:**
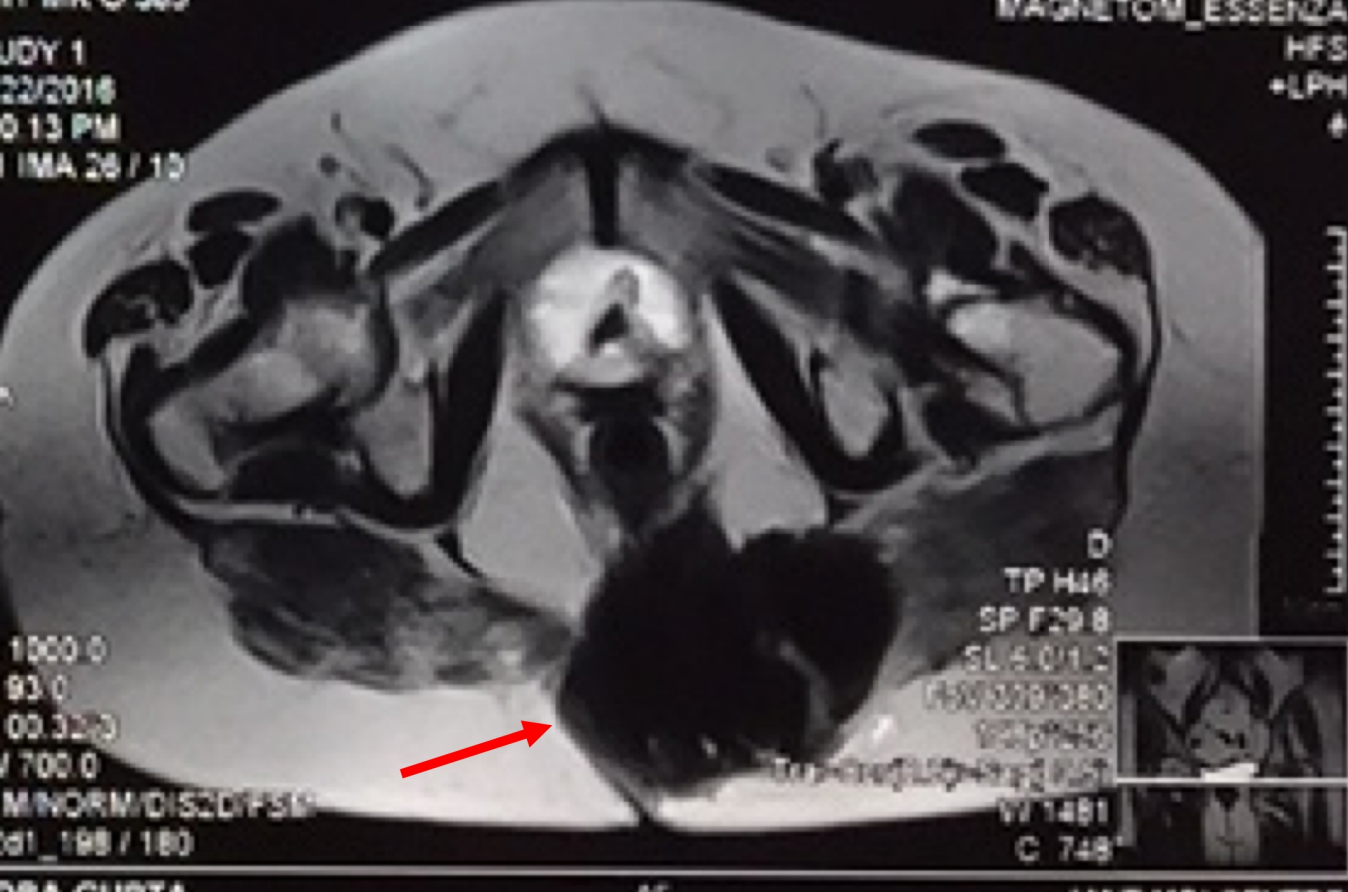
Computed tomography (CT) of the pelvis showing well defined heterogenous lesion in coccygeal region (red arrow) extending into pre- and postcoccygeal compartments in midline and left para midline 84 x 85 x 89 mm in dimension. Few foci of calcifications are noted.

A 77-year-old female presented to our outpatient department with a history of coccydynia for the past three months. The patient was initially thought of a simple coccydynia and was started on conservative treatment for coccydynia. However, the patient's symptoms were not relived and she complained of increasing pain which was present during the night also. On physical examination, an ill-defined mass of 10 x 8 cm approximately, hard in consistency was noticed in the coccygeal region. The mass was found to be fixed to the underlying structures and immobile. On per rectal examination, the mass could be felt in the left posterior quadrant which was hard in consistency and surface was found to be lobulated. The patient had no history of constitutional symptoms. Plain X-ray of the pelvis showed an ill-defined shadow of the mass with a corresponding destruction of the coccyx. Contrast computed tomography (CT) of abdomen and pelvis revealed a heterogenous lesion of soft tissue density in the midline and left paramedian region of the coccyx (extending into pre- and postcoccygeal plane) measuring 8.4 x 8.9 cm as seen in Figure 1.

Based on the appearance and location, the differential diagnosis included chordoma or a soft tissue sarcoma. The chest radiograph was normal. Biopsy was done, which showed diagnosis to be sclerosing epithelioid sarcoma. Positron emission tomography (PET) scan was done which showed no metastasis.

Operative details

**Figure 2:**
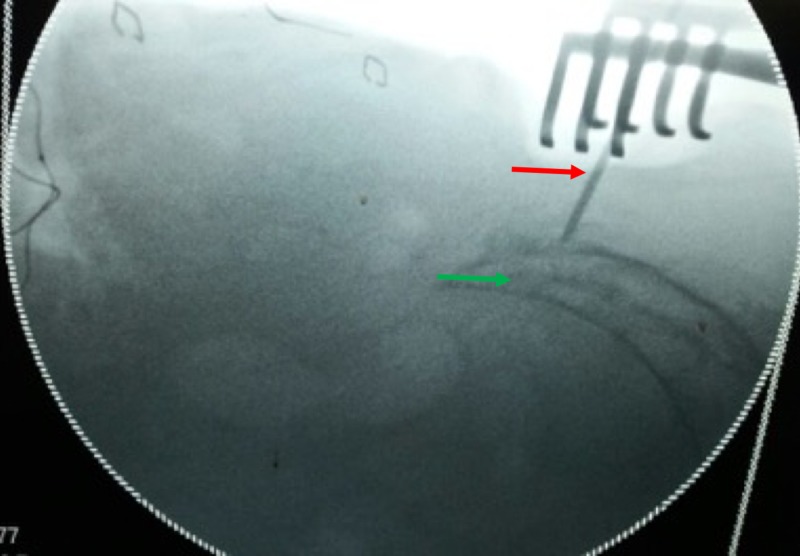
Radiological marking of the proximal extent of the tumor: Green arrow (Marked with a ‘K’ wire: Red arrow).

Following pre-anesthesia clearance, bowel was prepared to prevent fecal contamination in case of an unlikely event of a rectal perforation. In the prone position, the buttocks were retracted laterally with adhesive tape to expose the gluteal cleft. The coccygeal and anal regions were prepared with povidone-iodine. Lateral fluoroscopic image was used to locate the S3 level as shown in Figure 2.

**Figure 3:**
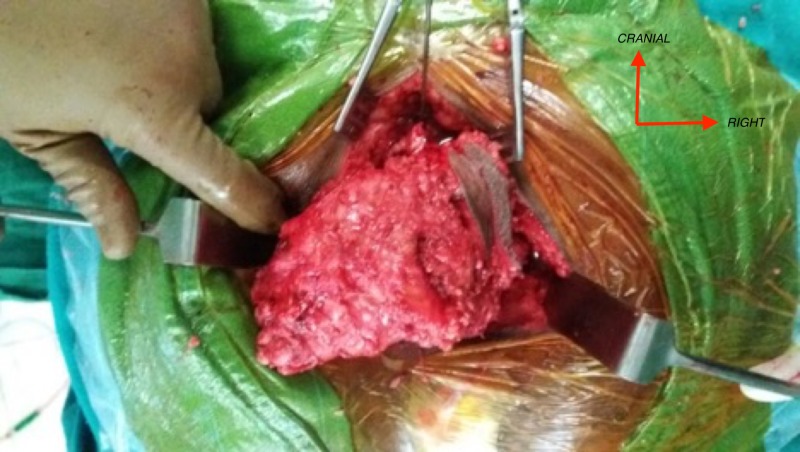
Intraoperative images of tumor resection. The tumor appears well circumscribed and measured 12.5 x 7.5 x 8.5 cm with attached pubococcygeal muscle.

A midline vertical incision is made from S3 till few centimeters caudal to the palpable mass. The tumor mass was separated from the surrounding tissues taking healthy margins circumferentially and separating it from skin and fascia laterally and from recto-sacral fascia ventrally taking care of the rectum. The tumor was removed en-bloc as seen in Figure 3 by resecting through the body of S4 vertebrae.

However, sacral nerves were spared. Hemostasis was secured and the wound was closed in layers over a drain. The patient is symptom-free at two years follow-up.

Pathological examination

**Figure 4:**
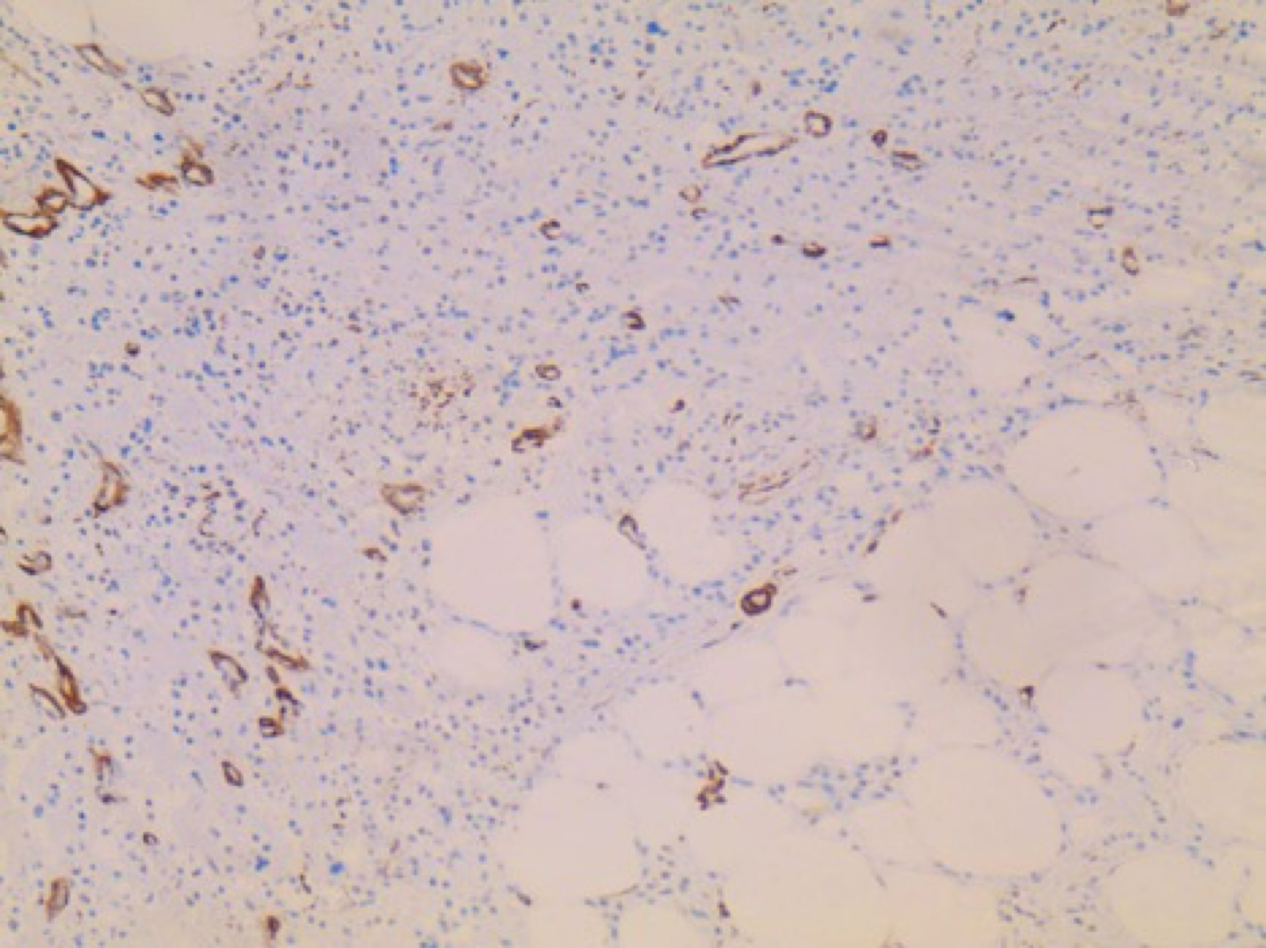
H&E staining of the mass. The tumor is composed of cells, which are rich in cytoplasm and show pale, vesicular, and irregular nuclei. The tumor cells are embedded in a collagen-rich extracellular matrix and display no signs of regression.

The resected specimen was of 12.5 x 8.5 x 7.5 cm and was submitted for pathological examination. The cut section was grey-white with minimal hemorrhages. Microscopic examination showed round to oval spindle-shaped epitheloid cells with mild pleomorphism embedded in eosinophilic collagenous bands of different thickness as seen in Figure 4.

Cells were arranged in files, cords, nests, vague whorled patterns, and sheets. Scant mitosis was seen without the presence of necrosis. Resection margins were free of infiltration. Immunohistochemistry was positive for vimentin and negative for other markers (SMA, CK, Desmin, CD 34, EMA, CD 45, S100, CD31, HMB45). The pathology examination was consistent with sclerosing epithelioid fibrosarcoma.

## Discussion

Sclerosing epithelioid fibrosarcoma is a very rare mesenchymal tumor, which mainly affects patients of middle age with equal gender predisposition. It was first described by Meis Kindblom and grouped under low-grade fibrosarcomas [2]. Being very slow growing, in most cases, it takes around 33 months from the onset of symptoms to the diagnosis. The tumor typically develops from deep musculature and often infiltrates adjacent fascia and periosteum. It has a predilection for local recurrence and metastasizes primarily to the lung. The mean interval between first diagnosis and the appearance of metastasis is around 7.7 years [2].

Our patient was elderly female with a relatively rapid onset of symptoms (three months). SEF typically involves the extremities or the torso and presents as a deep tissue mass [2]. The available literature describes these tumors in unusual locations too such as abdominal viscera [3,4], head and neck [5,6] and rarely arising as a primary tumor within bone [1]. In our case, due to the relatively superficial location of the tumor (coccyx), it became visually appreciable much earlier and presented as coccydynia.

Pathognomic histology finding includes epithelioid cells arranged in cords and nests in a background of dense collagenous extracellular matrix. SEF is likely to be mistaken for benign soft tissue lesions in lieu of their usual bland appearance including nodular fasciitis, fibrous histiocytoma, ossifying myositis or the hyalinizing leiomyoma. However, in lesions that demonstrate greater cytological atypia, the epithelioid appearance can lead to an improper diagnosis of metastatic carcinoma. With regard to intraosseous SEF, the dense collagenous matrix associated with the lesion can be misinterpreted as osteoid, leading to misdiagnosis as osteosarcoma [7-10].

Electronic microscopy reveals cells of ovoid or angular configuration, with fine chromatin, and well-formed nucleoli. The cytoplasm has characteristics similar to those of fibroblasts, with abundant rough endoplasmic reticulum. Immunohistochemical study is confirmatory. This study must include the determination of cytokeratins, epithelial membrane antigen, common leukocyte antigen, muscle-specific actin, protein S-100, neurospecific enolase, desmin, and vimentin. The most characteristic is vimentin which is positive in our case.

Due to the rarity of the condition no standard treatment protocols have been advocated. Moreover, preoperative or postoperative radiation as used in other soft tissue sarcomas also should be considered in cases which cannot be excised with clear margins.

To our knowledge, this is the first case of coccygeal sclerosing epithelioid fibrosarcoma. Due to lack of literature reporting SEF in coccyx region, it is not uncommon to confuse these tumors with chordoma arising from sacrum and surgeon should always keep in mind a differential diagnosis of SEF in tumors arising from coccyx.

## Conclusions

Sclerosing epithelioid fibrosarcoma is histologically low-grade sarcoma but clinically aggressive tumor. A differential diagnosis of SFE should be made in tumors arising from coccyx. All cases of coccydynia should not be treated as idiopathic and clinical; radiological examination should be done in all patients.
